# Bioinformatic Evaluation of Transcriptional Regulation of WNT Pathway Genes with reference to Diabetic Nephropathy

**DOI:** 10.1155/2016/7684038

**Published:** 2015-11-30

**Authors:** Gareth J. McKay, David H. Kavanagh, John K. Crean, Alexander P. Maxwell

**Affiliations:** ^1^Centre for Public Health, Queen's University Belfast, Belfast BT12 6BA, UK; ^2^Conway Institute, University College Dublin, Dublin 4, Ireland

## Abstract

*Objective.* WNT/*β*-catenin pathway members have been implicated in interstitial fibrosis and glomerular sclerosis disease processes characteristic of diabetic nephropathy (DN), processes partly controlled by transcription factors (TFs) that bind to gene promoter regions attenuating regulation. We sought to identify predicted *cis*-acting transcription factor binding sites (TFBSs) overrepresented within WNT pathway members. *Methods.* We assessed 62 TFBS motif frequencies from the JASPAR databases in 65 WNT pathway genes. *P* values were estimated on the hypergeometric distribution for each TF. Gene expression profiles of enriched motifs were examined in DN-related datasets to assess clinical significance. *Results.* Transcription factor AP-2 alpha (*TFAP2A*), myeloid zinc finger 1 (*MZF1*), and specificity protein 1 (*SP1*) were significantly enriched within WNT pathway genes (*P* values < 6.83 × 10^−29^, 1.34 × 10^−11^, and 3.01 × 10^−6^, resp.).* MZF1* expression was significantly increased in DN in a whole kidney dataset (fold change = 1.16; 16% increase; *P* = 0.03). *TFAP2A* expression was decreased in an independent dataset (fold change = −1.02; *P* = 0.03). No differential expression of *SP1* was detected. *Conclusions.* Three TFBS profiles are significantly enriched within WNT pathway genes highlighting the potential of *in silico* analyses for identification of pathway regulators. Modification of TF binding may possibly limit DN progression, offering potential therapeutic benefit.

## 1. Introduction

Diabetic nephropathy (DN) is a microvascular complication of diabetes and the most frequent cause of end-stage renal disease (ESRD) in western populations [1]. Approximately one-third of those with prolonged duration of diabetes will develop DN regardless of their glycemic control [2]. The earliest phase of DN is characterized by kidney hypertrophy and an increased glomerular filtration rate (GFR) with later progression resulting in pathological changes in the kidney including expansion of mesangial matrix, glomerular sclerosis, and interstitial fibrosis. Clinical features of DN include persistent proteinuria, hypertension, and progressive decline in GFR. ESRD secondary to DN necessitates costly renal replacement therapies, such as dialysis and renal transplantation. However, a subset of individuals with poorly controlled type 1 diabetes (T1D) do not develop DN [3]. A strong predisposition to DN exists with an increased estimated sibling risk (2.3-fold) supporting an underlying genetic susceptibility to this diabetic complication [4]. In addition, evidence in support of variation in ethnic genetic DN susceptibility has been previously reported [5].

**Figure 1:**
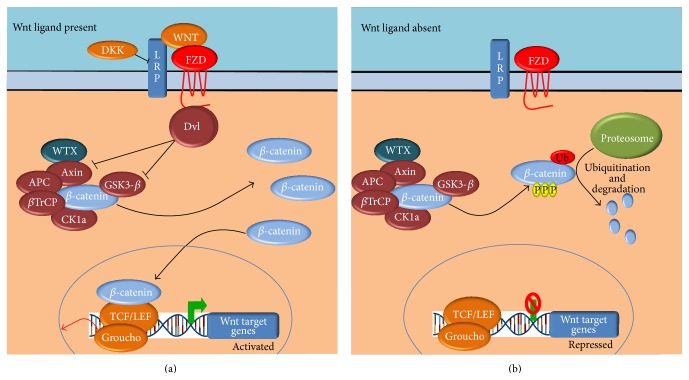
The canonical Wnt signalling pathway implicated in diabetic nephropathy. When a WNT ligand binds to the Frizzled (FZD) and the LRP5/6 coreceptors, the Dvl protein is recruited and inhibits the binding of *β*-catenin, leading to an increase in cytoplasmic *β*-catenin levels and subsequent activation of downstream signalling targets (a). In the absence of any WNT ligand (b), *β*-catenin is sequestered by the Axin complex containing such proteins as GSK3-*β*, APC, CK1, *β*TrCP, and axin itself. *β*-catenin is then phosphorylated and ubiquitinated and enters the proteasomal degradation pathway and leading to subsequent repression of downstream signalling targets.

Renal interstitial fibrosis and glomerular sclerosis are characteristic hallmarks of DN and several studies have implicated members of the WNT/*β*-catenin pathways in these disease processes [6–9]. The WNT pathways can be separated into canonical *β*-catenin dependent and noncanonical *β*-catenin independent pathways (Figure 1). Canonical WNT signalling is integral to many developmental processes and associated variants have been identified in multiple WNT pathway members with respect to many complex diseases such as familial adenomatous polyposis coli, colorectal and hepatocellular cancers, type 2 diabetes, and schizophrenia [10]. Noncanonical WNT signalling remains less well characterized, partly as a consequence of further subdivisions into the WNT/Ca^2+^ and the WNT planar cell polarity pathways. The WNT/*β*-catenin pathways have been reported to alter cytoskeletal reorganization and activation of the JNK and MAPK signalling [11, 12], directly affecting mesangial cell motility and adherence resulting in blunting of mesangial cell reaction to dynamic mechanical forces (a key mesangial cell function).

*In vitro* epithelial-to-mesenchymal transition (EMT) is induced by TGF-*β*1 [13], an integrin-linked kinase which promotes renal fibrosis. Both the canonical WNT pathway and TGF-*β*1 require activation of *β*-catenin implicating both *β*-catenin and the WNT pathway in the regulation of EMT [14]. In addition, the *β*-catenin phosphorylating protein, GSK3-*β*, is responsible for subsequent proteasomal degradation and has been reported to inhibit transition to a mesenchymal phenotype in human embryonic stem cells [15]. Differential gene expression profiles for several WNT ligands, FZD receptors, and *β*-catenin have been identified in the unilateral ureteral obstructed (UUO) mouse model of renal injury [6]. Independently, the WNT signalling antagonist, Dickkopf-1 (DKK1), was reported to promote hyperglycemia-induced matrix expansion in rat mesangial cells [7].

Differential gene expression of many developmental and pathological processes is partly controlled by transcription factors (TFs), proteins that bind to the promoter regions of genes affecting their transcription to mRNA [16]. Transcriptional regulation is modulated primarily by upstream elements in the DNA sequence. RNA polymerase II is directed to the transcription start site (TSS) by a series of “general transcription factors” (GTFs) necessary for* in vitro* transcription [17] which assemble approximately 25 to 30 base pairs (bp) upstream of the TSS and typically contain motifs such as the TATA box [18].

While the GTFs interact with a mediator forming a large complex to initiate transcription* in vivo* [19], additional influences are exerted by upstream elements located both proximally and distally from the TSS. Enhancer and repressor elements can initiate, amplify, reduce, or inhibit transcription of a given gene and various TFs bind to these regulatory elements [20]. The TFs are proteins or protein complexes that contain DNA-binding and activation domains which recognize specific sequence motifs and act on some target in the transcriptional machinery or the surrounding chromatin structure in order to modulate transcription [21]. A TF can recognize different sequence elements across many genes providing a mechanism for the coordinated expression of multiple genes or pathways in parallel by a single element.

Previously, we have assessed common genetic variants within key WNT pathway genes for association with DN [22, 23] and there is evidence that many WNT pathway genes are differentially regulated in the pathogenesis of DN [6–9]. In this study we sought to identify* cis*-acting regulatory elements in groups of coregulated genes by searching for an overrepresentation of known TF binding motifs within the promoters of WNT pathway genes and compare these to a background set of sequences, typically other gene promoters within the genome [24–26]. We used TF binding site (TFBSs) data from the JASPAR database [27] on 65 known WNT pathway genes to identify common transcriptional regulatory mechanisms associated with the WNT pathway.

Although current therapeutic options have been shown to reduce proteinuria and retard DN progression, recent studies highlight that, despite improved care, the higher risks of cardiovascular disease, ESRD, and mortality associated with DN persist [28]. As such, identification of genetic factors that may influence susceptibility to and development of DN can help identify novel pathophysiologic mechanisms as potential therapeutic targets to improve the adverse clinical outcomes that currently exist in diabetic patients. Despite several genome wide association studies to investigate common genetic variants and more recent identification of rarer variants though whole exome sequencing, the genetic architecture of DN remains poorly understood [3, 29, 30]. Given the low level of genetic variation associated with DN, we hypothesized if overrepresentation of TFBS motifs in WNT pathway gene members might influence genetic risk and offer future potential therapeutic target pathways.

## 2. Methods

Research ethics approval was obtained from the South and West Multicentre Research Ethics Committee (MREC/98/6/71) and Queens University Belfast Research Ethics Committee.

### 2.1. Identification of WNT Pathway Genes

We used the KEGG database which is a repository that stores pathway based information and “molecular circuit” maps to identify 65 targets for investigation [31].

### 2.2. Definition of Promoter Sequences and Identification of TFBSs

The JASPAR database (2010; http://jaspar.genereg.net/) catalogues 68 human TF position frequency matrices, although six were incomplete for the purpose of reconstructing position weight matrices using the programming language R. Upstream DNA sequence information for 65 WNT pathway genes was interrogated for recognized binding sites relating to the 62 annotated TFs documented in the JASPAR database amenable to analysis. Frequency correlations for observed TF motifs identified 5,000 bp upstream of WNT pathway genes were compared to ~28,000 gene sequences from genome build hg19/GCRh37.3 (http://hgdownload.cse.ucsc.edu/goldenPath/hg19/bigZips/upstream5000.fa.gz). *P* values for each TF were estimated on their hypergeometric distribution which describes the probability of *k* successes in *n* draws from a finite population without replacement. This distribution was used to model the probability of finding a TFBS more frequently in a chosen set of gene promoter sequences than in a set of random gene promoters alone [24].

### 2.3. Clinical Evaluation of Enriched Transcription Factor Expression Profiles

The clinical significance of gene expression profiles of enriched motifs was examined from several DN-related datasets. Nephromine combines a rapidly growing compendium of publicly available human renal gene expression profiles with a sophisticated analysis engine and an application for data mining and visualization of gene expression data (http://www.nephromine.org). The Schmid diabetes dataset (hereafter referred to as whole kidney) is characterized within Nephromine and comprises expression data from cDNA microarrays of whole kidney biopsies from healthy living donors (*n* = 3), cadaveric donors (*n* = 4), minimal change disease patients (*n* = 4), and DN donors (*n* = 11) [32]. The Woroniecka datasets in Nephromine comprise nine diabetic kidney disease (DKD) microdissected glomeruli analysed against thirteen control glomeruli and ten DKD tubulointerstitium and vascular compartments (hereafter referred to as tubulointerstitium) evaluated against twelve control tubulointerstitium samples [33]. Additional data examining the effect of the proinsulin C-peptide on the profibrotic actions of TGF-*β*1 [34] was obtained from the Gene Expression Omnibus (GEO) database. This data was generated from HK-2 cells (immortalised human proximal renal tubular cells [35]) for three control samples and three TGF-*β*1 (2 ng/mL) samples treated for 48 hours (hereafter referred to as HK-2 + TGF-*β*) and analysed using the gcrma R/bioconductor package for data normalization (http://www.bioconductor.org/packages/release/bioc/html/gcrma.html), with changes in expression estimated using the limma R/bioconductor package for the computation of fold change (http://www.bioconductor.org/packages/2.12/bioc/html/limma.html), test statistics, and *P* values. Expression levels, fold change, and significance were assessed for each TF.

## 3. Results

### 3.1. Motif Enrichment Analysis

**Figure 2:**
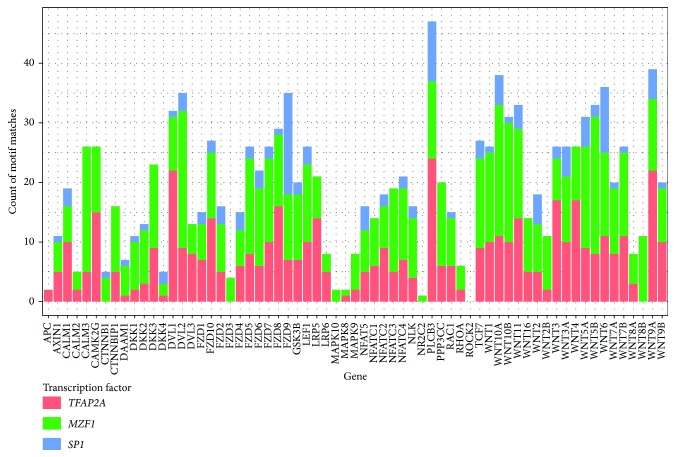
Total count for transcription factor binding site sequence motifs per WNT pathway gene. Red, green, and blue represent the counts for* TFAP2A*,* MZF1*, and* SP1*, respectively. Only those motifs identified with a confidence score greater than 95% were included.

**Figure 3:**
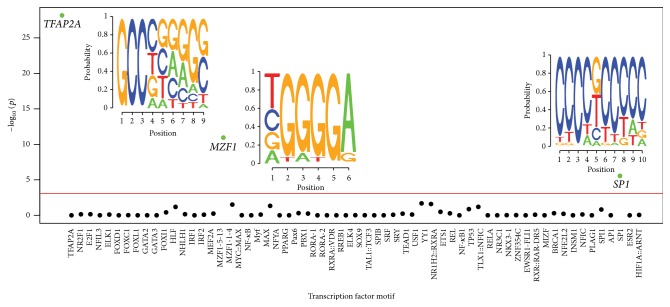
Enrichment analysis of JASPAR transcription factor binding sites (TFBSs). Putative TFBSs were evaluated in both the WNT pathway gene set and across the entire genome for enrichment using the hypergeometric distribution with Bonferroni correction for multiple testing (the red line indicates *P* = 0.001).* TFAP2A*,* MZF1*, and* SP1* were significantly enriched across WNT pathway genes (*P* < 6.83 × 10^−29^, 1.34 × 10^−11^, and 3.01 × 10^−6^, resp.). TFBS sequence motifs are illustrated with the height of each base indicative of the probability of the presence of the corresponding base at the designated position.

Motif enrichment analysis (*n* = 62) was completed on the promoter regions of 65 WNT pathway genes and ~28,000 NCBI documented genes as comparative controls, focusing on 5,000 bp regions upstream from the TSS. The total number of binding sites for each TF/gene was also calculated with only those motifs where the confidence score exceeded 95% included (Figure 2). A Bonferroni correction for multiple testing established a significance threshold level of *P* < 0.001 (*P* = 0.05/62). The TFBS motifs transcription factor AP-2 alpha (*TFAP2A*), myeloid zinc finger 1 (*MZF1*), and specificity protein 1 (*SP1*) were identified as significantly enriched within the WNT pathway dataset compared to the background gene set with *P* values estimated at 6.83 × 10^−29^, 1.34 × 10^−11^, and 3.01 × 10^−6^, respectively and are represented as probability sequence motifs (Figure 3).

**Figure 4:**
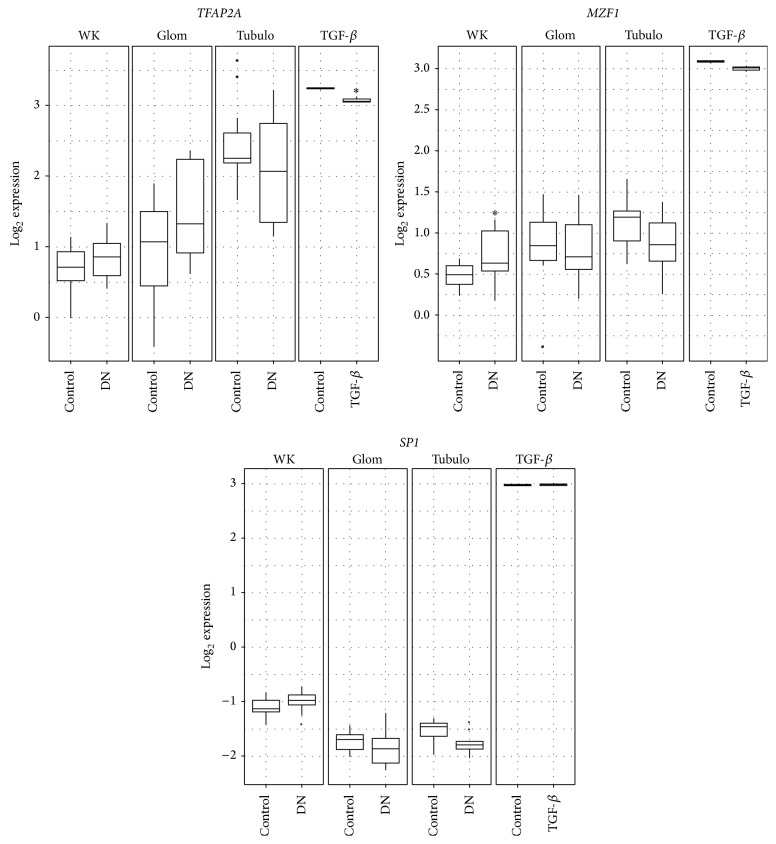
Expression of enriched transcription factors from four independent datasets. Log_2_ expression values evaluated in Nephromine (http://www.nephromine.org) from whole kidney (WK [32]) biopsies, glomerulus (Glom [33]) biopsies, tubulointerstitium (Tubulo [33]) biopsies, and from HK-2 cells treated with TGF-*β* for 48 hours (TGF-*β* [34]).* MZF1* gene expression was significantly increased in diabetic nephropathy (DN) whole kidney tissue compared to non-DN control (fold change = 1.16; *P* = 0.031).* TFAP2A* gene expression was decreased in HK-2 cells treated with TGF-*β* compared to untreated HK-2 cells (−1.02-fold change; *P* = 0.031).

Clinical evaluation of enriched transcription factor expression profiles showed significant increased gene expression of* MZF1* in DN in the whole kidney dataset with a fold change of 1.16 (16% increase, *P* = 0.03; Figure 4).* TFAP2A* gene expression was decreased in the HK-2 + TGF-*β* dataset with a fold change of −1.02 (*P* = 0.03; Figure 4).* SP1* did not show any differential expression in the datasets examined.

### 3.2. Pair-Wise Correlation of Motifs

**Figure 5:**
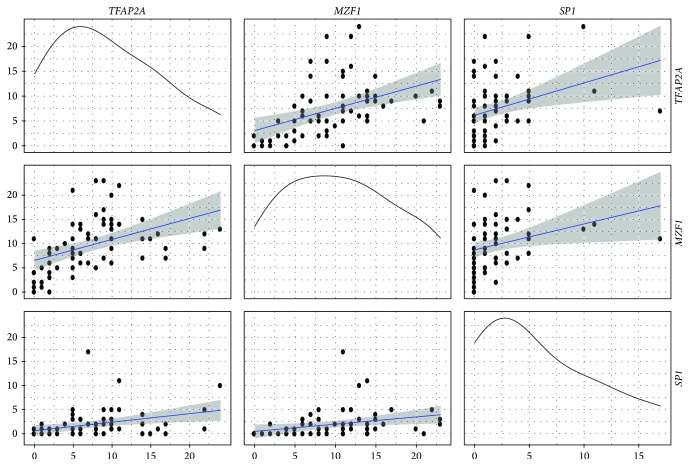
Correlation coefficients calculated between transcription factor binding sites (TBFSs) to estimate potential interaction and regulatory control of Wnt pathway genes. The number of binding sites identified for each motif was compared using Spearman's rank correlation. TFAP2A and MZF1 (*r* ^2^ = 0.59; *P* = 1.87 × 10^−7^); TFAP2A and SP1 (*r* ^2^ = 0.46; *P* = 1.09 × 10^−4^); MZF1 and SP1 (*r* ^2^ = 0.41; *P* = 6.26 × 10^−4^).

The total number of binding sites for each TF/gene was calculated (Figure 2) and Spearman's rank correlation test performed to estimate the correlation between the number of TFBSs identified and each TF examined (Figure 5). TFAP2A was more strongly correlated with MZF1 (*r* ^2^ = 0.59; *P* = 1.87 × 10^−7^) and less so with SP1 (*r* ^2^ = 0.46; *P* = 1.09 × 10^−4^). MZF1 was also significantly correlated with SP1 (*r* ^2^ = 0.41; *P* = 6.26 × 10^−4^).

## 4. Discussion

The* in silico* approach adopted in this study to assess transcription factor binding motif enrichment has predicted three TFBSs to be significantly enriched within the WNT pathway genes examined. These transcription factors have been reported previously in relation to cancer biology and other cellular processes involved in the pathogenesis of DN, such as regulating epithelial-to-mesenchymal transition, TGF-*β* signalling, and fibrogenesis. A common regulatory mechanism underpinning these processes with respect to DN may offer a promising potential therapeutic target. Hyperglycemia has been shown to downregulate WNT signalling resulting in increased TGF-*β* and fibronectin expression in glomerular mesangial cells. Induced upregulation of WNT4, WNT5a, and stabilization of cytosolic *β*-catenin have been reported to minimize the damaging effects of TGF-*β*1 induced fibronectin expression, in a manner similar to that observed through pharmacological inhibition of GSK3-*β* [8].

TFAP2A is a TF involved in the regulation of multiple developmental processes, such as neural crest formation and kidney development [36]. The activator protein-2 (TFAP2) family of transcription factors includes five closely related proteins TFAP2A-E [37]. Reduced expression levels of TFAP2A have been associated with increased metastatic capability in breast cancer [38] with poor prognosis reported in gastric adenocarcinoma patients [39]. Reduction of TFAP2A in extravillous trophoblasts reduces EGF-dependent invasion, as well as levels of MMP-2 and urokinase plasminogen activator, proteins involved in extracellular matrix degradation [40]. Polymorphic variants within TFAP2A have also been shown to interact directly with APC and *β*-catenin preventing *β*-catenin from associating with TCF4 and thus blocking transcription of WNT-responsive genes in colorectal cancer cells [41].

Myeloid zinc finger 1 (MZF1, also known as ZNF42) is a two-domain TF, with each domain containing four and nine zinc finger arrangements recognizing separate but similar sequences [42]. The motif found to be enriched in our study corresponds with the first four zinc finger domain. MZF1 plays a key role in embryonic stem cell hematopoietic differentiation, yet its canonical function involves regulation of genes associated with growth, differentiation, and apoptosis of cells during myeloid lineage [43]. Much like TFAP2A, MZF1 has been reported in relation to multiple cancers. In colorectal and cervical cancer cells overexpressed MZF1 was shown to induce migration and invasion. MZF1 has also been implicated in increased expression of PKC*α* in hepatocellular carcinoma with reported reduction in invasion, migration, and proliferation in these cells with MZF1 siRNA [44].

SP1 is reported to regulate many processes including expression of genes modulating angiogenesis, apoptosis, cell growth, differentiation, and immune response [45]. The functionality of SP1 is cell specific leading to different or even opposing roles depending on the cellular context. Treatment with TGF-*β* has been shown to reduce SP1 expression in human articulated chondrocyte cells but increases SP1 expression in skin cells [46, 47]. SP1 also leads to downregulation of TGF-*β*RI and TGF-*β*RII following treatment with TGF-*β* [47]. Fibroblasts treated with TGF-*β* have increased levels of SP1 and subsequently type 1 collagen synthesis. Subsequent blockade of SP1 induction leads to a reduced collagen response [46]. Zhang and colleagues [48] identified four SP1 binding sites in the putative promoter region of the adiponectin gene* ADIPOQ* (adipocyte C1q and collagen domain containing) providing evidence of reduced promoter transcriptional activity as a result of genetic variation. Regulation of genes, such as GAPDH, insulin-like growth factor, calmodulin, and PAI1 by insulin, has also shown to be, at least in part, mediated by SP1 [49].

A major limitation of this motif enrichment analysis is reflected in the relatively small number of human TFBS motifs represented in the JASPAR database (*n* = 62) which is limited to investigating the effects of* cis*-acting elements only to the exclusion of* trans*-acting factors. The human genome encodes numerous transcription factors, many of which remain unidentified and may potentially modulate genes involved in the WNT pathway directly or indirectly. Gene regulation at the transcriptional level is multifaceted with multiple epigenetic mechanisms such as DNA methylation and histone modification involved, further compounding the level of complexity. Nevertheless, the motif enrichment analysis of the 65 WNT genes in this study identified three motifs that were represented significantly more frequently among WNT pathway genes than across the genome and, as such, are likely to represent major regulatory mechanisms that govern the expression, activation, and functions of the WNT pathways.

TFAP2A and MZF1 have both been implicated in the regulation of genes that control tumour invasiveness and metastases and the pathological process of EMT is known to underpin many cancer types with evidence supporting its role in metastatic cancer cells [50]. Given the role of EMT in renal fibrosis and the putative role of the WNT pathways in the aetiology of DN, our data suggests a role for these transcription factors in the pathogenesis of DN. In addition, SP1's role in the regulation of TGF-*β* signalling and collagen production suggests an influence on the disease processes involved in DN. Improved transcriptional control mechanisms may offer potential therapeutic targets for the treatment of disorders such as DN, which may result as a consequence of aberrant WNT pathway mechanisms.

## 5. Conclusions

Our findings highlight the merit of utilizing* in silico* analyses for the prediction of TFBSs and key regulators of WNT pathway genes, particularly when considered in conjunction with gene expression data. Insights into the pathological processes and molecular mechanisms which contribute to the progression of DN have important therapeutic implications. Modifications of TF binding to promoter regions of genes involved in these processes have been shown to reduce the rate of DN progression in several models of diabetes [51]. Refinement of targeted therapeutic strategies to modify transcriptional control of disease processes will become possible through clearer delineation of their role.

## Abbreviations

DN: Diabetic nephropathy
ESRD: End-stage renal disease
TF: Transcription factor
TFBS: Transcription factor binding site
TSS: Transcription start site
GFR: Glomerular filtration rate
GTFs: General transcription factors
mRNA: Messenger ribonucleic acid
bp: Base pairs
HK2: Human proximal renal tubular cells
GEO: Gene Expression Omnibus
EMT: Epithelial-to-mesenchymal transition
ng/mL: Nanograms per millilitre.
